# Predominant east to west colonizations across major oceanic barriers: Insights into the phylogeographic history of the hydroid superfamily Plumularioidea, suggested by a mitochondrial DNA barcoding marker

**DOI:** 10.1002/ece3.5608

**Published:** 2019-10-02

**Authors:** Carlos J. Moura, Allen G. Collins, Ricardo S. Santos, Harilaos Lessios

**Affiliations:** ^1^ MARE‐IMAR‐OKEANOS Department of Oceanography and Fisheries University of the Azores Horta Portugal; ^2^ National Systematics Laboratory NOAA's National Marine Fisheries Service Smithsonian National Museum of Natural History Washington DC USA; ^3^ Smithsonian Tropical Research Institute Balboa Panamá

**Keywords:** amphi‐Atlantic dispersal, benthic invertebrates, glaciations, global warming, nonindigenous species, Tethys Sea

## Abstract

We provide preliminary insights into the global phylogeographic and evolutionary patterns across species of the hydrozoan superfamily Plumularioidea (Cnidaria: Hydrozoa). We analyzed 1,114 16S sequences of 198 putative species of Plumularioidea collected worldwide. We investigated genetic connections and divergence in relation to present‐day and ancient biogeographic barriers, climate changes and oceanic circulation. Geographical distributions of most species are generally more constrained than previously assumed. Some species able to raft are dispersed widely. Human‐mediated dispersal explains some wide geographical ranges. Trans‐Atlantic genetic connections are presently unlikely for most of the tropical‐temperate species, but were probably more frequent until the Miocene–Pliocene transition, before restriction of the Tethys Sea and the Central American Seaway. Trans‐Atlantic colonizations were predominantly directed westwards through (sub)tropical waters. The Azores were colonized multiple times and through different routes, mainly from the east Atlantic, at least since the Pliocene. Extant geminate clades separated by the Isthmus of Panama have predominantly Atlantic origin. Various ancient colonizations mainly directed from the Indian Ocean to the Atlantic occurred through the Tethys Sea and around South Africa in periods of lower intensity of the Benguela upwelling. Thermal tolerance, population sizes, dispersal strategies, oceanic currents, substrate preference, and land barriers are important factors for dispersal and speciation of marine hydroids.

## Graphical abstract

We use hundreds of DNA barcodes, with a calibrated “molecular clock” analysis of the hydroid superfamily Plumularioidea, to investigate phylogeographic patterns, and the processes likely underlying them, on a global scale. Our analysis uses ancestral state reconstructions for oceanic region to make inferences that help to explain both present and past biogeographic divergences and genetic interconnections, in relation to changes in climate and ocean circulation.

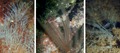

## 1 INTRODUCTION

Spatial distributions and evolutionary diversifications of marine species are constrained by biological/physiological adaptations to disperse and survive in different environmental conditions. In general, dispersal ability, temperature, productivity, oceanic circulation and habitat complexity and configuration are major determinants of population abundance and intra‐ and interspecific ecological interactions that promote diversification and control biogeographic ranges (reviewed by Bowen et al., 2016; Costello & Chaudhary, 2017).

For most marine benthic species, the nature and behavior of the pelagic stage of their life cycle, in conjunction with local hydrography, influences genetic dispersal and consequent geographical range limits (e.g., Scheltema, 1971; Todd, 1998; Underwood & Chapman, 1996). Larval development, duration, release timing, and behavior in the water column, among other characteristics of larval life, influence the distance of dispersal (e.g., Shanks, 2009; Siegel, Kinlan, Gaylord, & Gaines, 2003). Supposedly, short‐lived pelagic larvae disperse less far, constraining population connectivity and increasing genetic structure (Shanks, Grantham, & Carr, 2003). However, some organisms may also disperse to distant locations, taking advantage of anthropogenic transport (e.g., Carlton & Geller, 1993; Ruiz, Carlton, Grosholz, & Hines, 1997) or rafting on natural substrates (e.g., Cornelius, 1992a; Thiel & Gutow, 2005). Contemporary and historic patterns of oceanic circulation, physical barriers, and availability of suitable habitat, as well as ecological interactions and historic demography, will then affect the spatial genetic structure and speciation patterns of marine organisms (e.g., Ayre, Minchinton, & Perrin, 2009; Palumbi, 1994; Vermeij & Grosberg, 2010).

**Figure 1:**
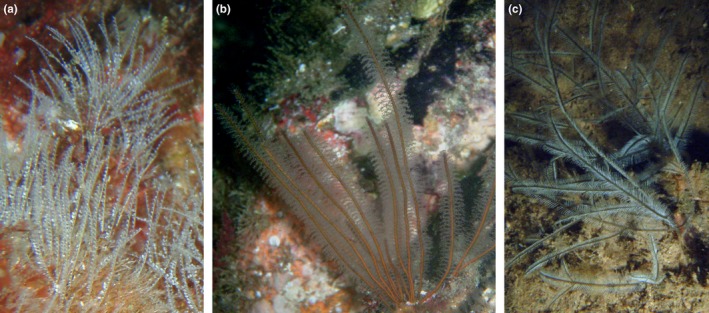
Examples of Plumularioidea hydroids thought to have trans‐Atlantic distributions: (a) *Antenella secundaria* (Halopterididae); (b) *Nemertesia antennina* (Plumulariidae); (c) *Macrorhynchia philippina* (Aglaopheniidae). *A. secundaria* and *N. antennina* are also thought to be present in both shallow and deep waters. Photograph scales are not uniform. Credits: Carlos J. Moura

Hydrozoans of the superfamily Plumularioidea (Figure 1) spend most of their life cycle attached to a substrate in the form of benthic polypoid colonies. They do not have a medusa planktonic phase; instead, colonies develop modified polyps (termed “fixed gonophores”) that produce gametes. Sperm are released to the water column, fertilization occurs inside female gonophores, and then a short‐lived planula larva with limited swimming ability is released, crawls up to a few centimeters and metamorphoses into a fixed polyp that develops into an upright colony via clonal reproduction without budding (e.g., Hughes, 1977; Postaire, Gélin, Bruggemann, Pratlong, & Magalon, 2017b; Sommer, 1992; Yund, 1990). A few species of Plumularioidea, however, may release medusoids to the water column (e.g., Bourmaud & Gravier‐Bonnet, 2004; Galea, Ferry, & Bertot, 2012; Migotto & Marques, 1999; Motz‐Kossowska, 1907). These medusoids are nonfeeding and short‐lived rudimentary medusa‐like organelles that liberate gametes into the water column. Although a few species produce planktonic medusoids, spatial proximity is required for fertilization, so long‐distance dispersal should be highly constrained for even these species of Plumularioidea. However, some hydroids may disperse through opportunistic rafting on other organisms (e.g., algae, crustaceans) or natural or anthropogenic materials (e.g., wood, plastics and boats). The superfamily Plumularioidea is highly speciose (Bouillon, Gravili, Pagès, Gili, & Boero, 2006) and characterized by patterns of high genetic structure of populations and species (Moura, Cunha, Porteiro, Yesson, & Rogers, 2012; Moura et al., 2018; Postaire, Gelin, Bruggemann, & Magalon, 2017a; Postaire, Gélin, et al., 2017b; Schuchert, 2014). These characteristics, along with the frequent presence of plumularioids in benthic environments worldwide, both in shallow and deep waters, make these hydroids interesting subjects for the investigation of phylogeographic patterns and historical processes in oceanic settings.

Taking advantage of 1,114 available DNA barcodes of Plumularioidea hydroids (16S mtDNA sequences; cf. Moura et al., 2018), we investigated phylogeographic and evolutionary patterns within the superfamily. Our sampling is particularly rich for extant Plumularioidea from parts of the N Atlantic and NE Pacific, which we use to calibrate a “molecular clock”, aiming to link genetic lineage divergence with changes of climate, continental drift, and oceanic circulation. The present study thus provides the first comprehensive insights on the global phylogeographic history of the hydroid superfamily Plumularioidea in the Cenozoic Era.

## 2 METHODS

**Figure 2:**
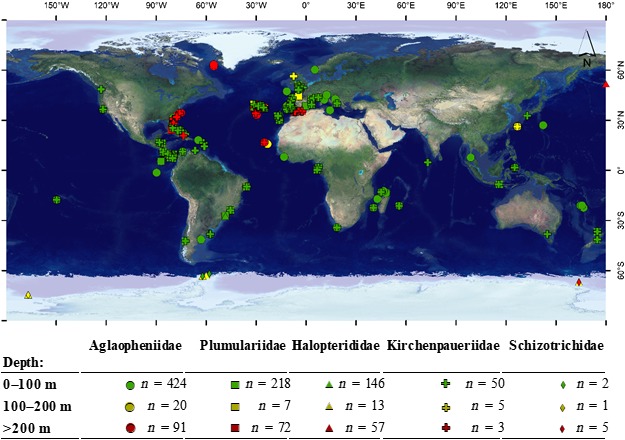
Geographical location and depth range of DNA sequences used in this study for each Plumularioidea family

The present study uses 1,114 16S sequences of different Plumularioidea, collected from various depths and locations worldwide (see sampling data and Genbank accession numbers in Table S1; Figure 2). Twenty‐one nominal genera are represented: one of Schizotrichidae (the only nominal genus of that family), eight (out of 13 nominal genera) of Aglaopheniidae, three (out of six genera) of Kirchenpaueriidae, five (out of 15 nominal genera) of Halopteriidae, and four (out of eight nominal genera) of Plumulariidae. The Plumularioidea were identified morphologically into 123 nominal species and 17 unknown (likely new) morphospecies, but species delimitation analyses of Moura et al. (2018) proposed a total of 198 putative species: 80 of Aglaopheniidae; seven of Schizotrichidae; 11 of Kirchenpaueriidae sensu stricto (s.s.); 48 of Halopterididae s.s.; and 52 of Plumulariidae s.s. The present analyses use the biodiversity units proposed by the species delimitation analyses of Moura et al. (2018). Three 16S sequences of Sertulariidae served as outgroup to the full DNA sequence dataset. See sequence data in Table S1.

### 2.1 Phylogenetic analyses

DNA sequences were entered in Geneious R10.0.2. The different datasets of sequences analyzed included all sequences of Plumularioidea together and sequences of each family separately. DNA sequences were aligned with MAFFT (Katoh, Misawa, Kuma, & Miyata, 2002). Subsequently, for the datasets with sequences separated by families, we used the Gblocks server (Castresana, 2000) with relatively strict settings (excluding gapped positions and minimizing the number of contiguous nonconserved positions) to obtain alignments (alignments were as in Moura et al., 2018; see Table S1 of the “Supplementary Information” of ref Moura et al., 2018).

**Figure 3:**
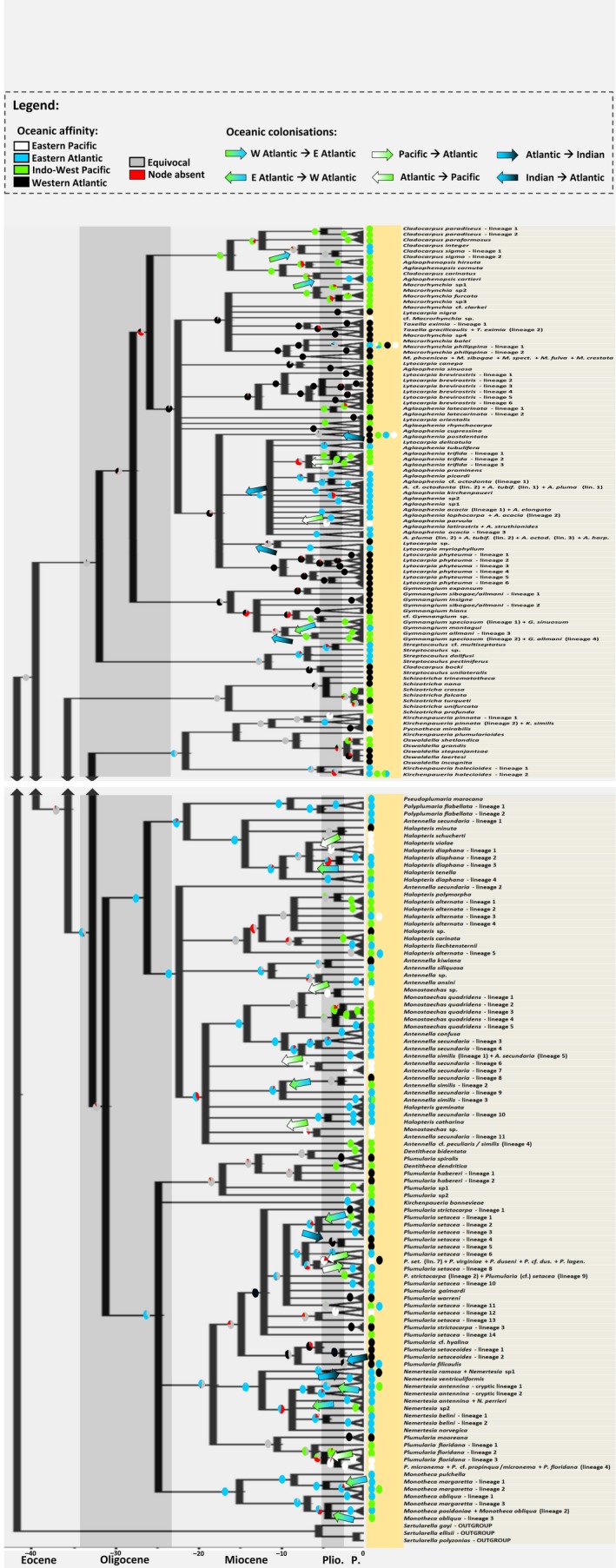
Time‐calibrated phylogeny of the hydroid superfamily Plumularioidea. Nodes collapsed presented posterior probabilities below 70%. Lineages were subsumed by putative species as determined by Moura et al. (2018). Circles at nodes indicate relative proportion of ancestral states (oceanic affinity) given by the ASR analyses: white—East Pacific; green—West Atlantic; blue—East Atlantic; black—Indo‐West Pacific; gray—equivocal; red—node absent). Arrows indicate probable direction of colonization between oceanic regions: turned left means eastward colonization, turned right westward colonization. See Figure S2 for complete results of the ASR analyses

Phylogenetic reconstructions included maximum likelihood (ML) and Bayesian inference (BI) tree searches for all the generated alignments. The general time‐reversible model of nucleotide evolution with gamma and invariant parameters (GTR + G+I) was used for all analyses, after assessment of the most suitable model using the “Akaike Information Criterion.” ML analyses were conducted in PhyML (Guindon & Gascuel, 2003) (version 20120412), with 1,000 bootstrap iterations. Bayesian analyses were performed with MrBayes v.3.2.2 (Ronquist, Teslenko, & Mark, 2012) and consisted of two runs of four chains each of 100 million generations with trees sampled every 1,000 generations after a burn‐in fraction of 25% of the trees (as in Moura et al., 2018). A summary of the nodal support obtained with the different ML and Bayesian analyses with the different sequence datasets is shown in Figure S1 and was contrasted with the “Ancestral state reconstruction” results presented in Figure 3 and Figure S2.

To estimate divergence times, we applied the Bayesian relaxed dating implemented in BEAST v.1.8.3 (Drummond, Suchard, Xie, & Rambaut, 2012), for the dataset with all the 16S sequences of Plumularioidea with indels present. We used a Yule speciation process prior for rates of cladogenesis (Drummond & Rambaut, 2007) in combination with an uncorrelated lognormal clock. As calibration points, we used clades presumed to have diverged due to the Central American Seaway closure ca. 3 Mya (e.g., Lessios, 2008); see subsection “Connectivity between the Atlantic and Eastern Pacific”). Thirty‐seven runs ranging from 63.5–100 million generations each (average = 92.4 millions), with every 10,000 generations sampled, were performed on the online computer cluster CIPRES Science Gateway (Miller, Pfeiffer, & Schwartz, 2010). Tracer v. 1.6 (Rambaut, Suchard, Xie, & Drummond, 2014) was used to check convergence of parameter estimates and ESS values. LogCombiner v1.8.3 (Drummond et al., 2012) was used to combine tree files from the multiple runs, resampling states every one million generations and using a burn‐in of 2,500 for each run. TreeAnnotator v1.8.3 (Drummond et al., 2012) was used to produce a consensus tree.

### 2.2 Ancestral state reconstructions (ASR)

Likelihood‐based ASR were conducted in Mesquite v.3.2 (Maddison & Maddison, 2017). Characters (East Pacific = 0, East Atlantic = 1, West Atlantic = 2, Indo‐West Pacific = 3) were traced over the tree resulting from analyses performed with BEAST (Drummond et al., 2012). To account for topological uncertainty, we used the “trace character over trees” option, which summarizes the ASR over a series of trees. All reconstructions were integrated over 5,699 trees, obtained with LogCombiner v1.8.3 (Drummond et al., 2012) after combining the thirty‐seven runs obtained from BEAST, and resampling states at a lower frequency of 600,000, using a burn‐in of 2,200 for each run. As a model of evolution for the ML reconstructions, we employed the Markov k‐state 1 (Mk1) parameter model, with equal probability for any particular character change.

## 3 RESULTS AND DISCUSSION

The large number of 16S sequences of Plumularioidea used in this study provided particularly good representation of shallow and deep‐water taxa of the N Atlantic (Figure 2). Similarly, relatively strong sampling in the Indo‐Pacific, especially for the families Aglaopheniidae and Plumulariidae, permitted an initial understanding of phylogeographic relationships between oceans. We use the “molecular clock” of Plumularioidea to assess hypotheses regarding approximate epochs of splitting of lineages, in relation to past climatic, geological, and oceanographic changes. We use the term “colonization” to denote that the extant species is found in a location different than the one inferred to be occupied by its ancestor.

### 3.1 Evolutionary relationships over time

Figure 3 shows a time‐calibrated phylogeny of the Plumularioidea suggested by 16S sequence data. The extant Plumularioidea families most likely originated by the Paleogene/ early Neogene. Our phylogeny places the root and early divergences of Plumularioidea during the middle/end of the Eocene, when oceans started to cool after a major global warming period (e.g., Alexander, Meissner, & Bralower, 2015; Lear, Bailey, Pearson, Coxall, & Rosenthal, 2008; Pearson & Palmer, 2000; Zachos et al., 2005). A great part of the extant genera of the Plumularioidea probably persisted through the warm temperatures and climate disruptions of the middle Miocene (Figure 3; e.g., Zachos, Pagani, Sloan, Thomas, & Billups, 2001; Retallack, 2002), which induced extinctions in other groups (e.g., Sepkoski, 1992,). The great diversification of extant lineages started mainly thereafter, coinciding with the subsequent general cooling of oceans. The high levels of cladogenesis observed since the last third of the Miocene, notably observed in Plumularioidea from NE Atlantic, match with the great constriction of the Tethys/Paratethys Seaway (Hüsing et al., 2009; Popov et al., 2006), followed by the rise of the Isthmus of Panama (Lessios, 2008), which led to major changes in oceanic circulation and climate (Butzin, Lohmann, & Bickert, 2011; Hamon, Sepulchre, Lefebvre, & Ramstein, 2013; Mudelsee & Raymo, 2005). Such events likely induced divergence of populations, but also extensive species radiations (similar to the history of cetaceans as reported by Steeman et al., 2009), as suggested for example by the radiation of *Aglaophenia* species of the NE Atlantic (Figure S1).

Numerous lineage divergences occurred through the (Plio‐)Pleistocene (Figure 3), as they did in other marine life (Ludt & Rocha, 2015; Pinheiro et al., 2017), when high origination rates of biodiversity likely counterbalanced high extinction rates (Allmon, Rosenberg, Portell, & Schindler, 1993). Interestingly, Antarctic clades, of *Schizotricha* and *Oswaldella*, probably radiated into a number of species in a short amount of time (Pleistocene‐Pliocene; Figure S1), possibly due to colonizations after deglaciation(s), at a time of increased Southern Ocean surface water productivity and elevated circum‐Antarctic temperatures (Cook et al., 2013).

### 3.2 Trans‐Atlantic genetic connectivity

#### 3.2.1 Recent trans‐Atlantic dispersal

Recent or ongoing trans‐Atlantic dispersal is only suggested for four Plumularioidea species: *Macrorhynchia philippina*, *Kirchenpaueria halecioides*, *Halopteris* cf. *alternata* (“lineage 5”), and *Antennella similis* (“lineage 3”) (Figure 3). These are the only putative species that shared the same 16S haplotype between the E and W Atlantic. Nevertheless, more haplotype sampling, both in studied and little studied regions (e.g., cold NW Atlantic and S Atlantic), may reveal the presence of more haplotypes shared across the Atlantic, as well as other phylogeographic patterns.

*Kirchenpaueria halecioides* exhibits the same haplotype in Argentina, Madeira, Azores, and mainland Portugal, mostly in ports and marinas (including on a ship hull). This taxon is thus likely to disperse via boats to remote locations. Remarkably, the eight other haplotypes of *K. halecioides*, also mainly collected from ports or marinas, are not so widely dispersed (each one collected once). This suggests that the haplotype found simultaneously at eight sampling stations may be the best adapted for adverse/diverse abiotic conditions associated with boat travel and to different destinations.

*Macrorhynchia philippina* has a widespread haplotype occurring in W Africa, Brazil, the Caribbean, and the E Pacific, suggesting also dispersal mediated by boats. Two other haplotypes of that taxon are widely distributed across the Indo‐Pacific and Indian Ocean, respectively. Besides boat traffic, the wide distribution of *M. philippina*, may also be explained by the liberation of medusoids as part of its life cycle, which could aid dispersal via oceanic currents, and its large population's sizes in warm waters.

*Halopteris* cf. *alternata* (“lineage 5”) has small sessile colonies and is frequently an epibiont on algae, sponges, and rocks. Although it seems to not release medusoids, the rafting abilities of this taxon (e.g., perhaps on algae) may explain the presence of two haplotypes with amphi‐Atlantic distribution, with one of these haplotypes also present in the central E Pacific. Although this taxon was not found in marinas or ports, we do not discard the hypothesis of its transport via boats as well.

*Antennella similis* (“lineage 3”) forms small colonies collected from a wide variety of substrates, including rock, dead coral, sponges, and fishing lines. Therefore, rafting on natural of artificial substrates probably also explains its recent trans‐oceanic dispersal.

That no other Plumularioidea were represented by shared haplotypes between the E and W Atlantic suggests that presently trans‐Atlantic genetic connections are unlikely for most of these benthic suspension feeders, not even through rafting (except on boats). This agrees with the general pattern of present‐time restricted trans‐Atlantic genetic connections observed in most studies of marine invertebrates (e.g., Ilves, Huang, Wares, & Hickerson, 2010; Nunes, Norris, & Knowlton, 2011; Pálsson, Magnúsdóttir, Reynisdóttir, Jónsson, & Örnólfsdóttir, 2014), and also with only sporadic observations of marine invertebrates (e.g., Claremont, Williams, Barraclough, & Reid, 2011; Nunes et al., 2011) or fishes (e.g., Bowen, Bass, Muss, Carlin, & Robertson, 2006) that show haplotypes with amphi‐Atlantic distributions.

A few other (putative) species contain genetically close haplotypes on both sides of the Atlantic, suggesting relatively recent trans‐Atlantic genetic connections, namely *Aglaophenia postdentata*, *Nemertesia* cf. *antennina* (”cryptic lineage 1”), *Plumularia* cf. *setacea* (“lineage 11”), and *Monotheca* cf. *margaretta* (“lineage 2”). The latter two taxa are frequent epibionts (e.g., Calder, 1997) and may have crossed the Atlantic by rafting facilitated by the strong and warm South Equatorial Current (Lumpkin & Garzoli, 2005). The observation of *Nemertesia* “cf. *antennina*” in deep waters of the west and east North Atlantic is surprising; large population sizes and/or an unidentified deep‐sea current may explain the amphi‐Atlantic distribution of this species. The geographical range of *Aglaophenia postdentata* on both sides of the Atlantic, central E Pacific, and Indian Ocean discovered by Moura et al.(2018) is intriguing because this species had been recorded only from the Indo‐Pacific and the Caribbean (Galea, 2010). Despite its wide geographical range, *A. postdentata* is rare, but perhaps its observed occasional growth on algae could have contributed to dispersal through rafting.

#### 3.2.2 Ancient trans‐Atlantic dispersal

All five detected cases of past trans‐Atlantic colonizations through tropical/subtropical shallow waters seem to have progressed in a westward direction (Figure 3; note arrows with blue and green colors), similar to what has been observed for other taxa (e.g., Baarli et al., 2017; Espinosa, Morey‐Rubio, & Nakano, 2016; Lapègue et al., 2002; Rocha et al., 2005; Vermeij, 1997; Vermeij & Rosenberg, 1993; Wangensteen, Turon, Pérez‐Portela, & Palacín, 2012) and in conformity with the predominant westward surface equatorial currents during the Neogene (Harzhauser, Piller, & Steininger, 2002; Vermeij, 2012; Vermeij & Rosenberg, 1993). It is not in agreement, however, with the predominantly eastward dispersal discovered in some fishes (Beldade et al., 2009; Floeter et al., 2008; Muss, Robertson, Stepien, Wirtz, & Bowen, 2001) and mollusks (Vermeij & Rosenberg, 1993). Our molecular dating analyses (Figure 3) suggest that these trans‐Atlantic genetic interconnections ceased around the end of the Miocene or in the Pliocene (e.g., in agreement with Baarli et al., 2017; Vermeij, 1997). This timing coincides with the constriction of the Central American Seaway (CAS) and Indonesian Gateway, which followed that of the Tethys Sea, leading to the end of the global equatorial surface current, global cooling and change of the internal circulation of Atlantic waters (Gravier, 1970; von der Heydt & Dijkstra, 2005; Karas et al., 2017; Keigwin, 1982; Landau, Silva, & Vermeij, 2015; Silva, Landau, & La Perna, 2011). These oceanographic changes might have thus promoted vicariance between the two sides of the Atlantic, ceasing the trans‐Atlantic distribution of many warm‐water species.

Regarding trans‐Atlantic dispersal through deep waters, there were two instances of past genetic connections in *Nemertesia* lineages from the east to western Atlantic that likely took place around the end of the Pliocene (Figure 3). Conversely, two past colonization events with reverse direction, that is, from the west to the east Atlantic, are suggested in the deep‐water clade of *Cladocarpus* and *Aglaophenopsis* (Figure 3). Although the direction of dispersal is inconclusive in representatives of *Cladocarpus sigma*, the divergence between *Cladocarpus carinatus* and *Aglaophenopsis cartieri* suggests a colonization from the NW to the NE Atlantic through deep waters (Figure 3), perhaps only up to the mid‐Atlantic ridge, where *A. cartieri* seems to be endemic (Ramil & Vervoort, 1992). Analogously, Eilertsen and Malaquias, (2015) discovered one gastropod species present in deep waters of the NW Atlantic and Azores, with lineages also separated around the Miocene–Pliocene transition. In fact, all the trans‐Atlantic vicariant events by deep waters represented by our data seem to have occurred roughly around the Miocene–Pliocene transition (Figure 3), coinciding with the intensification of the thermohaline circulation and with the formation of the North Atlantic deep water (e.g., Billups, 2002; Karas et al., 2017; Keigwin, 1982; Wright, Miller, & Fairbanks, 1991).

#### 3.2.3 Colonization of the Azores

The importance of seawater temperature for dispersal is evidenced by the Azorean fauna (Cornelius, 1992b), located close to the middle of the North Atlantic, having greater phylogeographic affinities with temperate/subtropical faunas of the NE Atlantic (namely, Macaronesian islands and seamounts, European shores and margins, and the Mediterranean), both in shallow and deep waters. The contemporary faunal composition suggests a reduced influence of the Gulf Stream for the Azores. The Gulf Stream cools as it travels from the Caribbean to the Azores, so that rafting hydroids may not survive this trip.

We find in several shallow‐water species (e.g., for *Aglaophenia picardi*, *Aglaophenia pluma* species complex, *Kirchenpaueris pinnata*, *Halopteris diaphana*, *Antenella secundaria*, and *Plumularia setacea*) multiple colonization events of the Azores from the east, at distinct times and through different routes (Figure S1). These westward colonizations seem more plausible after the Pliocene (Figure S1), coinciding with the reopening of the Mediterranean after the Messinian Salinity Crisis (e.g., Garcia‐Castellanos et al., 2009), when eddies originating in the vicinity of the Mediterranean and moving to west (Sala, Harrison, & Caldeira, 2015) probably became more frequent facilitating gene flow. This time also coincides with dramatic fluctuations of sea level due to glaciation episodes (e.g., Raymo, Ruddiman, Backman, Clement, & Martinson, 1989; Sosdian & Rosenthal, 2009), which may have facilitated stepping‐stone dispersal across Macaronesian islands and seamounts.

Notably, for those species that appear to have colonized the Azores through deep waters (e.g., of *Aglaophenia tubulifera*, *A. lophocarpa*, *Lytocarpia myriophyllum*, and *Polyplumaria flabellata*), the majority of the Azorean lineages cluster together, segregated from lineages of other localities (Figure S2). Additionally, the genetic diversity of these species (plus *Antennella confusa*) observed in the archipelago, especially in deep waters, is notable (Figure S1); perhaps a consequence of large population sizes and of the variety of habitats provided by the seamounts of the area.

In terms of dispersal strategy, rafting on algae or other floating animals or detritus should explain the presence of many of the shallow‐water hydroids in the Azores (as already hypothesized by Cornelius, 1992b); this is a dispersal method consistent with the multiple colonization events verified across various taxa (see paragraph above; Figure S2). Some hydroids have clearly arrived to the archipelago attached to boats, as is the case for *Kirchenpaueria halecioides*. The arrival of hydroids through deep waters should have been mostly through large or spatially close reproducing populations dispersed by gamete and larva liberation, carried by oceanic currents.

### 3.3 Connectivity between the Atlantic and Eastern Pacific

#### 3.3.1 Migrations across Central America

Only two of the species represented in this study—*Macrorhynchia philippina* and *Halopteris* cf. *alternata* (lineage 5), exhibit the same 16S haplotypes in the Atlantic and E. Pacific, which suggests recent or ongoing genetic interconnections between these oceanic basins. These taxa reveal preference for warm/tropical waters and present amphi‐Atlantic distributions as well (cf. last section). We therefore suspect these two species crossed the Isthmus of Panama with the aid of boats and/or rafting on other floating or swimming substrates (see Discussion on their dispersal strategies in the last subsection).

Eleven geminate clades of Plumularioidea were probably separated with the rise of the Isthmus of Panama, since the middle/end of the Miocene until the completion of that barrier by the Pliocene (see in Figure 3 lineages with white and green circles; e.g., Lessios, 2008). Excluding the clades mentioned in the previous paragraph for which we suspect human‐mediated transport, we used this geological event for the molecular clock calibrations the following clades: *Aglaophenia postdentata*; *A. trifida* + *A. prominens*; *Halopteris diaphana* “lineage 1”; *Halopteria alternata* “lineage 3”; *Monostaechas* spp.; *Plumularia setacea* “lineage 12”; *Plumularia floridana* complex. These sister taxa were collected from shallow waters of the Pacific and Caribbean coasts of Panama and/or Costa Rica, and share close morphological and genetic similarities, suggesting that they diverged at a time close to the completion of the Isthmus ca. 3 Mya. The following two lineages probably migrated across the Central American Seaway but were excluded from “molecular clock” calibrations because their respective sister groups on the Caribbean side were not sampled or readily apparent in the phylogenetic results by phylogenetic analyses: *Antennella “secundaria* lineages 6 and 7” and *Antennella “secundaria* lineage 11” + *Monostaechas* sp. Our analyses suggest the majority of genetic colonizations across the Central American Seaway were from the Atlantic to the Pacific (Figure 3, note the leftward direction and positioning of the arrows with white and green colors), in accordance with the hypothetical main westward surface flow of seawater until the completion of the Isthmus (e.g., Butzin et al., 2011; von der Heydt & Dijkstra, 2005; Heydt & Dijkstra, 2006; Motoi, Chan, Minobe, & Sumata, 2005) and colonization patterns verified in a great variety of other taxa (e.g., Groves, 1997; Kronenberg & Lee, 2007; Roopnarine, 2001; Waller, 2007); reviewed by Leigh et al.(Leigh, O'Dea, & Vermeij, 2013). We do not discard also the suggestions of eastward flow across the CAS, before the seaway closure (Miocene to Early Pliocene), followed by the western Atlantic “dramatic extinctions during and after formation of the isthmus” (as reviewed by Leigh et al., 2013), considering the lack of Plumularioidea fossils and extant genetic lineages that could support that complementary hypothesis. Nevertheless, the predominant westward colonization across the CAS we note for the Plumularioidea hydroids fits with the general westward pattern of trans‐Atlantic colonizations we verified until the Miocene restriction of the circum‐tropical surface current, as discussed in the subsection “Trans‐Atlantic genetic connectivity”.

#### 3.3.2 Migrations around the southern tip of South America

Our data only display one evident example of colonization across the seaway between South America and Antarctica, represented by a cold‐water clade of *Plumularia* species (“lineage 7”, plus the nominal species *P. duseni*, *P. virginiae*, and *P. lagenifera*) sampled from W. USA, Chile, Argentina, and New Zealand. That transition occurred probably from the south Pacific to the south Atlantic possibly before the early‐middle Pleistocene (Figure 3), when glacial episodes likely induced lineage divergence. Interestingly, prior to that event, this clade of *Plumularia* likely reached the E Pacific through the NW Atlantic via a trans‐Artic route (see below). Furthermore, that clade is apparently absent from the warm waters of the E. Pacific, presenting an antitropical distribution along the cold shallow waters of the E. Pacific, which reflects the sensitivity of these invertebrates to seawater temperature.

#### 3.3.3 Migrations through the trans‐Arctic route

Our analysis reveals three examples of cold‐water lineages of Plumularioidea that likely crossed N America through a trans‐Artic route: (a) *Plumularia setacea* from the Atlantic versus the cryptic cold‐water *Plumularia* present in the eastern Pacific; (b) *Kirchenpaueria pinnata* from the Pacific versus Atlantic; and (c) *Aglaophenia latirostris* plus *A. struthionides* from California versus cluster with *A. lophocarpa* and *A. parvula*. These interoceanic genetic interchanges likely took place around the Miocene–Pliocene transition (Figure 3), in accordance with dates provided by fossil taxa, between ca. 5.3 and 3.5 Ma (Gladenkov, Oleinik, Marinkovitch, & Baranov, 2002; Vermeij, 1991, 2004). These trans‐Artic Plumularioidea colonizations were probably from the Atlantic to the Pacific (Figure 3), which contradicts the prevalent eastward gene flow direction assumed for most marine taxa (e.g., Briggs & Bowen, 2013; Durham & MacNeil, 1967; Marincovich & Gladenkov, 1999; Vermeij, 1991; Vermeij, 2004) but agrees with inferred mollusk colonizations and predominant southward currents through the Bering Strait when it opened ca. 5.5–4.8 Mya (Marincovich, 2000; Marincovich, Barinov, & Oleinik, 2002). However, due to an underrepresentation of Plumularioidea samples from the temperate/cold waters of the NW Atlantic, we cannot exclude the hypothesis of postglacial recolonization of the NW Atlantic from the NW Pacific.

### 3.4 Connectivity between the Atlantic and Indian Ocean

#### 3.4.1 Migrations around South Africa

We did not find any Plumularioidea haplotype occurring simultaneously in the Atlantic and Indian Ocean (Figure 3). This suggests that presently genetic connections between these oceans are unlikely, possibly due to the effect of the Benguela upwelling system (e.g., Jury & Brundrit, 1992), combined with the limited thermal tolerance of Plumularioidea hydroids. Nevertheless, the present analysis suggests some Plumularioidea had maintained genetic contact between the Atlantic and Indian Oceans, either around the southern tip of Africa or through the ancient Tethys Sea (Figure 3). Our sampling captured four likely examples of Plumularioidea hydroid colonizations around Cape Agulhas, probably before the Pliocene–Pleistocene (Figure 3), when the Benguela upwelling started to intensify, albeit with some fluctuations of intensity (e.g., Diester‐Haass & Schrader, 1979; Marlow, Lange, Wefer, & Rosel‐Melé, 2000; Rommerskirchen, Condon, Mollenhauer, Dupont, & Schefuss, 2011; Shannon, 1985; Siesser, 1980). Our results suggest three colonization events from the Indian Ocean to the Atlantic rounding the southern tip of Africa, similar to other taxa (e.g., Briggs & Bowen, 2013; Floeter et al., 2008; Vermeij & Rosenberg, 1993), and one likely colonization in the reverse direction (Figure 3; Figure S2).

The split of *Nemertesia ramosa* collected from the NE Atlantic and Mediterranean, in relation to *Nemertesia* sp. from Japan, seems to have occurred by the end of the Miocene –Pliocene (Figure 3; Figure S2). By then, the Tethys Sea was already closed; thus, the divergence of these two clades is explained by the intensification of the Benguela upwelling system. This hypothesis agrees with the geographical distribution of *N. ramosa* in the eastern Atlantic through South Africa, and in the Indian Ocean in Mozambique (Ansín Agís, Ramil, & Vervoort, 2001). Our analyses suggest that in this case, colonization possibly occurred from the Atlantic to the Indian Ocean.

*Aglaophenia postdentata* seems to have an Indo‐Pacific origin, with apparent colonization at (at least) two independent times to the Atlantic, because lineages from the SW Indian Ocean cluster independently from lineages from Central West Africa and from the Caribbean, respectively (Figure 3; Figure S2). Such transitions likely occurred around Cape Agulhas, before the Pleistocene, when the intensity of the Benguela upwelling and Agulhas Current experienced cyclic fluctuations (e.g., Cornelius, 1992a; Rocha et al., 2005; Shannon, Lutjeharms, & Nelson, 1990; Vermeij & Rosenberg, 1993).

*Plumularia filicaulis* is represented by two lineages, one from Australia the other from the Atlantic side of South Africa, with the latter probably derived from the Indo‐Pacific. Such vicariance seems to have occurred by the early Pleistocene (Figure 3). This species did not reach further north in the Atlantic (e.g., Watson & Wells, 1997), thus not surpassing the biogeographic barrier caused by the upwelling of the cold Benguela current. An hypothesis is that this taxon might have dispersed through surface currents of the thermohaline circulation connecting Australia with South Africa.

#### 3.4.2 Ancient migrations through the Tethys Sea

We found four probable examples of genetic colonization through the Tethys Sea, prior to its closure around the Middle Miocene (see in Figure 3 clades with circles and arrows with black and blue colors; e.g., Harzhauser et al., 2002; Vrielynck, Odin, & Dercourt, 1997; Harzhauser et al., 2007). Furthermore, our results suggest these colonization events occurred from the Indian Ocean toward the Atlantic (Figure 3), a similar pattern as in fishes (Briggs & Bowen, 2013), consistent with modeling studies that suggest predominant water flow from the Indian to the Atlantic Oceans through the proto‐Mediterranean (e.g., Herold, Huber, Müller, & Seton, 2012; von der Heydt & Dijkstra, 2005). In contrast, Vermeij (2001) mentions invading clades of mollusks from tropical America and the European part of Tethys, toward the present Indian Ocean, before the Miocene.

The most interesting colonization between the Atlantic and Indian Ocean is represented by the clade containing Atlantic species of *Aglaophenia* (e.g., *A. tubulifera*, *A. picardi*, *A. lophocarpa*, *A. pluma*) that derived clearly from the Indo‐Pacific and radiated extensively in the Atlantic into several species after the closure of the Tethys Sea (Figure 3). Again, this may be evidence of the effect of the colonization of a new oceanic basin (i.e., the Atlantic), possibly with low biodiversity levels prior to that time, that likely experienced dramatic change in its oceanographic conditions after the closure of the Tethys Sea (e.g., Butzin et al., 2011; Hamon et al., 2013).

The Japanese clade containing *Cladocarpus bocki* and *Streptocaulus unilateralis* likely diverged by the Middle‐end Miocene from the clade containing deep water *Streptocaulus* spp. present in the Mediterranean and NE Atlantic (Figure 3), coinciding with the closure of the Tethys Sea. Similarly, the divergence of the ancestor of the NE Atlantic and Mediterranean *Lytocarpia miryophyllum* from its Indian Ocean dates at a similar timing, coincident with the closure of the Tethys Sea (Figure 3).

Atlantic *Gymnangium* also clearly derived from the Indo‐Pacific, but due to low support for some phylogenetic relationships between some clades of that genus, we cannot infer the route that the ancestors of *Gymnangium speciosum* (lineage 1) and *G. sinuosum* likely took to colonize the Caribbean Sea. However, an ancestor of *Gymnangium montagui* probably colonized the NE Atlantic from the Indian Ocean through the Tethys Sea; and subsequent colonizations occurred to the Caribbean side resulting in *Gymnangium allmani* (lineages 3 and 4) and *Gymnangium speciosum* (“lineage 2”) (Figure 3).

### 3.5 Connectivity across the Eastern Pacific Barrier (EPB)

Although we lack a comprehensive sampling in the Pacific, we have evidence that extant Plumularioidea from the E Pacific have higher affinities with the Atlantic instead of the W Pacific fauna (Figure 3), likely due to the effect of the East Pacific Barrier (e.g., Baums, Boulay, Polato, & Hellberg, 2012; Cortés et al.,; Darwin, 1872; Ekman, 1953). Nevertheless, there are a few examples of E Pacific lineages with sisters in the W Pacific, that seem to have crossed the EPB at different times through different routes, reflecting the sporadic permeability of this barrier (in agreement with other studies, e.g., Cowman & Bellwood, 2013; Duda & Kohn, 2005; Lessios & Baums, 2017; Lessios & Robertson, 2006).

A tropical clade of *Plumularia*, including *P. mooreana* and the cluster with morphotypes similar to *P. floridana*, seems to have crossed the EPB, possibly from west to east prior to the middle‐end of the Miocene (Figure 3). Conversely, temperate/cold‐water lineages of *Plumularia* spp. (clade with samples from California, Chile, Argentina, and New Zealand) and *Halopteris* spp. (clade with *H. minuta* from New Zealand and *H. schucherti* from Chile) seem to have crossed the EPB through cold waters of the South Pacific (eventually Antarctica), possibly from east to west, prior to the Pliocene–Pleistocene (Figure 3).

### 3.6 Dispersal strategies

The majority of the Plumularioidea (cf. Bouillon et al., 2006) disperse only through the release to the water column of gametes and short‐lived planula larvae without much swimming capability (e.g., Hughes, 1977; Hughes, 1986; Postaire, Gelin, et al., 2017a; Postaire, Gélin, et al., 2017b; Yund, 1990), and occasionally through rafting (e.g., Calder 1995; Cornelius, 1992a, 1992b) or sporadic detachment of (pieces of) colonies from their substrate. Consequently, and as noted (Figure S1), these hydroids exhibit great population subdivisions often related to physical distance, which tends to increase with the decrease of organismal abundances locally (because close proximity of colonies is often required for successful sexual reproduction).

*Macrorhynchia philippina*, *Gymnangium hians*, *Dentitheca bidentata*, and *Monotheca obliqua* are the only Plumularioidea among those represented in our study known to release medusoids (e.g., Bourmaud & Gravier‐Bonnet, 2004; Migotto & Marques, 1999; Motz‐Kossowska, 1907; Ronowicz et al., 2017). On its face, this would appear to be advantageous for large‐scale dispersal. Indeed, these species appear to have relatively wide distributions: *Macrorhynchia philippina* is circum‐(sub)tropical (e.g., Figure 3, Ansín Agís et al., 2001); *Gymangium hians* is widely distributed across the Indo‐Pacific (e.g., Figure 3; Ronowicz et al., 2017); *D. bidentata* is found off Brazil and by the E and W Africa (Migotto & Marques, 1999); and *M. obliqua* is considered cosmopolitan (Watson, 2011); although cryptic diversity may be associated (Moura et al., 2018). However, although medusoid liberation may facilitate longer dispersal by local water currents, medusoids when reared in the laboratory lived for only a few hours and were negatively buoyant, which might inhibit large‐scale dispersal but enhance fertilization success (e.g., Bourmaud & Gravier‐Bonnet, 2004; Gravier, 1970; Migotto & Marques, 1999). In fact, excepting *M. philippina*, we did not encounter shared haplotypes of these species from distant localities. Thus, medusoid liberation does not necessarily lead to large‐scale dispersal. Indeed, within the athecate hydroid family Hydractiniidae, lineages without feeding medusae generally present wider global distributions than species presenting feeding medusa stage (Miglietta & Cunningham, 2012).

*Macrorhynchia philippina* presents a phylogeographic structure similar to other Plumularioidea (Figure S1), conforming to generalized seawater circulation patterns. However, this species has been able to overcome hard and permeable barriers to dispersal easier than all other plumularioids (Figure 3). *Macrorhynchia philippina*, possibly with Indo‐Pacific origin (Figure 3), likely takes advantage of boat traffic for dispersal, as it was sampled near ports (Moura et al., 2018) and from artificial substrates like shipwrecks and is also associated with ropes (Riera, Espino, & Moro, 2016) and found in very shallow depths (Ansín Agís et al., 2001). Additionally, this species releases medusoids (Gravier, 1970), has a high concentration of nematocysts that provide protection, and develops prominent colonies (pers. obs.), allowing for large population sizes and high sexual fecundity.

*Kirchenpaueria halecioides* and *Halopteris* cf. *alternata* (lineage 5) are the other species represented with widely distributed haplotypes (Figure 3), probably as a result of dispersal by boat traffic. It is worth mentioning that “lineage 2” and “lineage 6” of *Plumularia setacea*, with samples collected in marinas, probably dispersed also through human‐mediated transport but across smaller geographic ranges. Some other nonplumularioids are also reported to take advantage on boat traffic for dispersal, such as those of genera: *Cordylophora* (e.g., Folino, 2000), *Pennaria* (e.g., Miglietta, Odegard, Faure, & Faucci, 2015), *Blackfordia* (e.g., Kramp, 1958), *Turritopsis* (Miglietta & Lessios, 2008), and *Clytia* (Calder et al., 2019).

*Aglaophenia postdentata*, despite our failure to detect widely dispersed haplotypes, seems to present a circum‐tropical distribution, with relatively small genetic distances between populations of various oceanic areas. Only this species and *M. philippina* were found simultaneously in the Indo‐Pacific and W and E Atlantic. Because *A. postdentata* was not detected near ports or marinas, we do not suspect human‐mediated dispersal. Although it appears to have small population sizes at present, its ability to overgrow algae suggests that rafting assisted by oceanic currents is the most plausible explanation for its large‐scale dispersal. However, we know little about its life cycle, and it is possible that medusoid release or long‐lived planula larvae could be discovered for this species.

Finally, reproduction and population sizes, and ultimately dispersal, are also likely to be affected by the growth mode of colonies, which impact the number of reproductive structures produced (Moura et al., 2012). For example, *Nemertesia ramosa* has large population sizes both in shallow and deep waters, and develops robust ramified colonies from which several reproductive structures can originate, favouring genetic connections across wide bathymetrical ranges. Inversely, demonstrating the correlation between hydroid growth and food availability (Di Camillo et al., 2017), “true” *Nemertesia antennina* develops prominent colonies in shallow waters only, leading to wide dispersal of haplotypes throughout the NE Atlantic and Mediterranean, whereas cryptic *Nemertesia* “*antennina*” has delicate colonies with few reproductive structures in deep waters, resulting in reduced geographic ranges of haplotypes and ultimately sympatric and parapatric speciation. The vulnerability or adaptability of species to thrive in different biotic and abiotic conditions, therefore, seems to affect population sizes and dispersal.

## 4 CONCLUSIONS

With a large (growing) dataset of DNA barcodes and “molecular clock” calibrations, we may discover many phylogeographic and evolutionary patterns. Nevertheless, the present study is exploratory and a generator of phylogeographic hypotheses, which apply to marine benthic invertebrates. Much of marine genetic diversity (including that of the Plumularioidea) remains little sampled/investigated; but with an increased number of DNA Barcoding and genomic studies, we will gradually increase our understanding of marine evolutionary history. To complement this particular study, it is desirable to sample more DNA barcodes from other oceanic settings, such as the southern and northwest Atlantic, and much of the cold waters of the Pacific and the deep sea. Furthermore, as other (combinations of) genes may suggest discordant phylogeographic patterns and thus more fine‐scale complex movements of genetic lineages, the usage of further genetic markers will also increase robustness of evolutionary inferences.

The major constraints to dispersal and speciation we hypothesized were dispersal strategy (e.g., especially rafting or not, but perhaps also with or without medusoids), population sizes, water temperature, and consequent tolerance of the marine fauna, coupled with the effects of oceanic circulation and land barriers. For example, vicariance induced by the closures of the Tethys Sea and Central American Seaway, as well as by glacial periods and oceanic current modifications, seems to have promoted considerable diversification of hydroids. Additionally, the Indo‐West Pacific seems to have played a major role as a source of genetic diversity to the Atlantic. Indeed, the majority of Atlantic species of Plumularioidea seem to have derived evolutionarily from the Indian Ocean, and from species radiations inside the Atlantic. We found some lineages that colonized the Atlantic via the ancient Tethys Sea. The maritime passage rounding South Africa served predominantly for westward genetic connections between the Atlantic and Indian Ocean, in periods of less activity of the Benguela current.

More recently, trans‐Atlantic migrations seem unlikely for most Plumularioidea hydroids of tropical to temperate waters. Haplotypes with amphi‐Atlantic distribution, suggestive of recent genetic interconnections across the Atlantic, were only detected in four hydroid species that likely disperse through rafting on natural and/or human‐made floating substrates. However, various tropical taxa migrated across the Atlantic in the past, mainly from east to west, taking advantage of equatorial currents. The main genetic break of trans‐Atlantic colonizations probably occurred around the Miocene–Pliocene transition, coinciding with the constriction of the Tethys Sea and the Central American Seaway, and consequent change of oceanic current velocities and other properties. Deep‐water clades of the N Atlantic seem to have mostly crossed the Atlantic from east to west, but with some exceptions.

Presently, the Gulf Stream does not seem to contribute much to trans‐Atlantic dispersal of Plumularioidea hydroids. The Azores, in the middle of the North Atlantic and greatly influenced by the Gulf Stream, is instead more phylogeographically related with the Mediterranean, Lusitanian province and Macaronesian islands and seamounts. The archipelago has been colonized multiple times and through different routes from the eastern Atlantic side, since the Pliocene to (close to) the present, sometimes more than once for a single species. Stepping‐stone dispersal across Macaronesian seamounts and islands, as well as eddies and deep‐sea currents departing from the vicinity of the Mediterranean, may explain the occurrence of Plumularioidea hydroids in the Azores. Few species arrived at the archipelago with the aid of boats. We displayed evidence of one lineage that reached the mid‐Atlantic ridge from the West Atlantic, but via deep waters.

Most (extant) geminate clades of Plumularioidea separated by the rise of the Isthmus of Panama have Atlantic origin. The few examples of colonizations across the ancient trans‐Artic route also suggest primarily colonizations from the Atlantic to the Pacific. To the south, however, Pacific fauna migrated to the SW Atlantic (eventually also in the reverse direction) using the passage between South America and Antarctica. The “Eastern Pacific Barrier” prevented higher phylogeographic influence of tropical West Pacific fauna, in the East Pacific and Atlantic. Nevertheless, a few lineages overcame that barrier, at different times and adopting different routes and directions, revealing the sporadic permeability of the barrier.

## CONFLICT OF INTEREST

The authors declare no competing financial and nonfinancial interests.

## AUTHOR CONTRIBUTIONS

The present work makes part of the postdoctoral research of C.J.M., supervised by R.S.S., A.G.C. and H.L. C.J.M. performed most of the analyses, under the supervision of H.L. C.J.M., H.L. and A.G.C. drafted the original manuscript. All authors read, corrected and approved the final version of the text.

## Data Availability

Supplementary files available at https://doi.org/10.5061/dryad.p33659q
