# Supplementary figures and images for: Predominant east to west colonizations across major oceanic barriers: Insights into the phylogeographic history of the hydroid superfamily Plumularioidea, suggested by a mitochondrial DNA barcoding marker
Source: Ecol Evol. 2019 Oct 2;9(23):13001–16. doi: 10.1002/ece3.5608 (PMC6912911; doi:10.1002/ece3.5608)

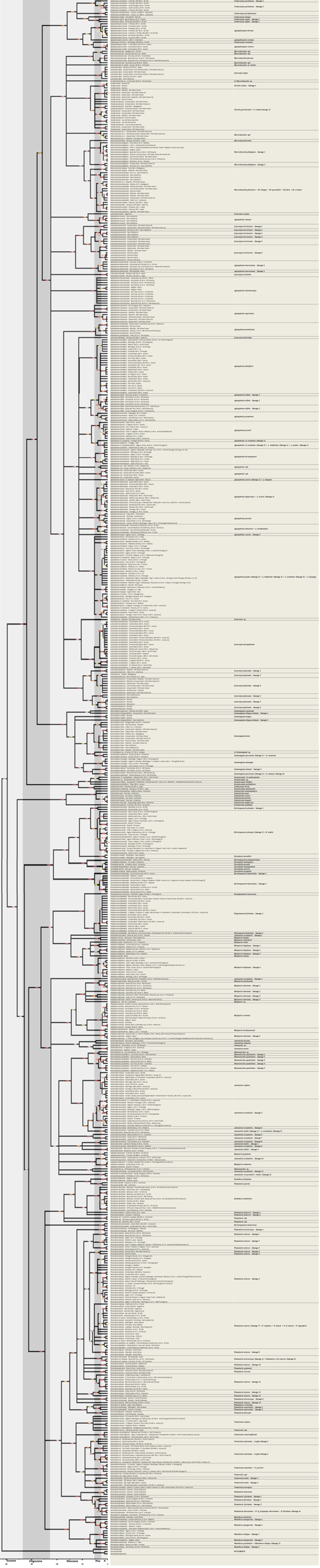

Supplement: Supplementary file 1 [file ECE3-9-13001-s001.pdf]
